# Biological effects of bergamot and its potential therapeutic use as an anti-inflammatory, antioxidant, and anticancer agent

**DOI:** 10.1080/13880209.2023.2197010

**Published:** 2023-04-17

**Authors:** Sabrina Adorisio, Isabella Muscari, Alessandra Fierabracci, Trinh Thi Thuy, Maria Cristina Marchetti, Emira Ayroldi, Domenico Vittorio Delfino

**Affiliations:** a Foligno Nursing School, University of Perugia, Foligno, Italy; b Department of Medicine and Surgery, Section of Pharmacology, University of Perugia, Perugia, Italy; c Bambino Gesù Children’s Hospital, IRCCS, Rome, Italy; d Institute of Chemistry, Vietnam Academy of Science and Technology Cau Giay, Graduate University of Science and Technology, Ha Noi, Vietnam

**Keywords:** *Citrus bergamia*, ethnomedicine, flavonoid, polyphenolic fraction of bergamot, antiproliferative, pro-apoptotic

## Abstract

**Context:** Bergamot, mainly produced in the Ionian coastal areas of Southern Italy (Calabria), has been used since 1700 for its balsamic and medicinal properties. Phytochemical profiling has confirmed that bergamot juices are rich in flavonoids, including flavone and flavanone glycosides which are responsible for its beneficial effects.

**Objective:** Recently, it was shown that the combination of natural compounds with conventional treatments improves the efficacy of anticancer therapies. Natural compounds with anticancer properties attack cancerous cells without being toxic to healthy cells. Bergamot can induce cytotoxic and apoptotic effects and prevent cell proliferation in various cancer cells.

**Methods:** In this review, the antiproliferative, pro-apoptotic, anti-inflammatory, and antioxidant effects of bergamot are described. Information was compiled from databases such as PubMed, Web of Science, and Google Scholar using the key words ‘bergamot’ accompanied by ‘inflammation’ and, ‘cancer’ for data published from 2015–2021.

**Results:** *In vitro* and *in vivo* studies provided evidence that different forms of bergamot (extract, juice, essential oil, and polyphenolic fraction) can affect several mechanisms that lead to anti-proliferative and pro-apoptotic effects that decrease cell growth, as well as anti-inflammatory and antioxidant effects.

**Conclusions:** Considering the effects of bergamot and its new formulations, we affirm the importance of its rational use in humans and illustrate how bergamot can be utilized in clinical applications. Numerous studies evaluated the effect of new bergamot formulations that can affect the absorption and, therefore, the final effects by altering the therapeutic profile of bergamot and enhancing the scientific knowledge of bergamot.

## Introduction

Chemical substances isolated from plants have played a central role in medicine by forming the basis for the development of many fundamental drugs. For example, in oncology, paclitaxel, and other plant compounds have been used as models for the design of synthetic drugs. Medicines isolated from plants are included in what is termed traditional medicine (TM). According to the World Health Organization (WHO), TM ‘refers to the knowledge, skills, and practices based on theories, beliefs, and experiences of an indigenous culture used to maintain health and prevent, diagnose, or treat physical and mental illness. TM spans a range of diverse therapies and practices that vary by country and region, including drugs composed of herbs, animal extracts, and/or minerals’ (Adorisio et al. 2016).

**Figure 1.**
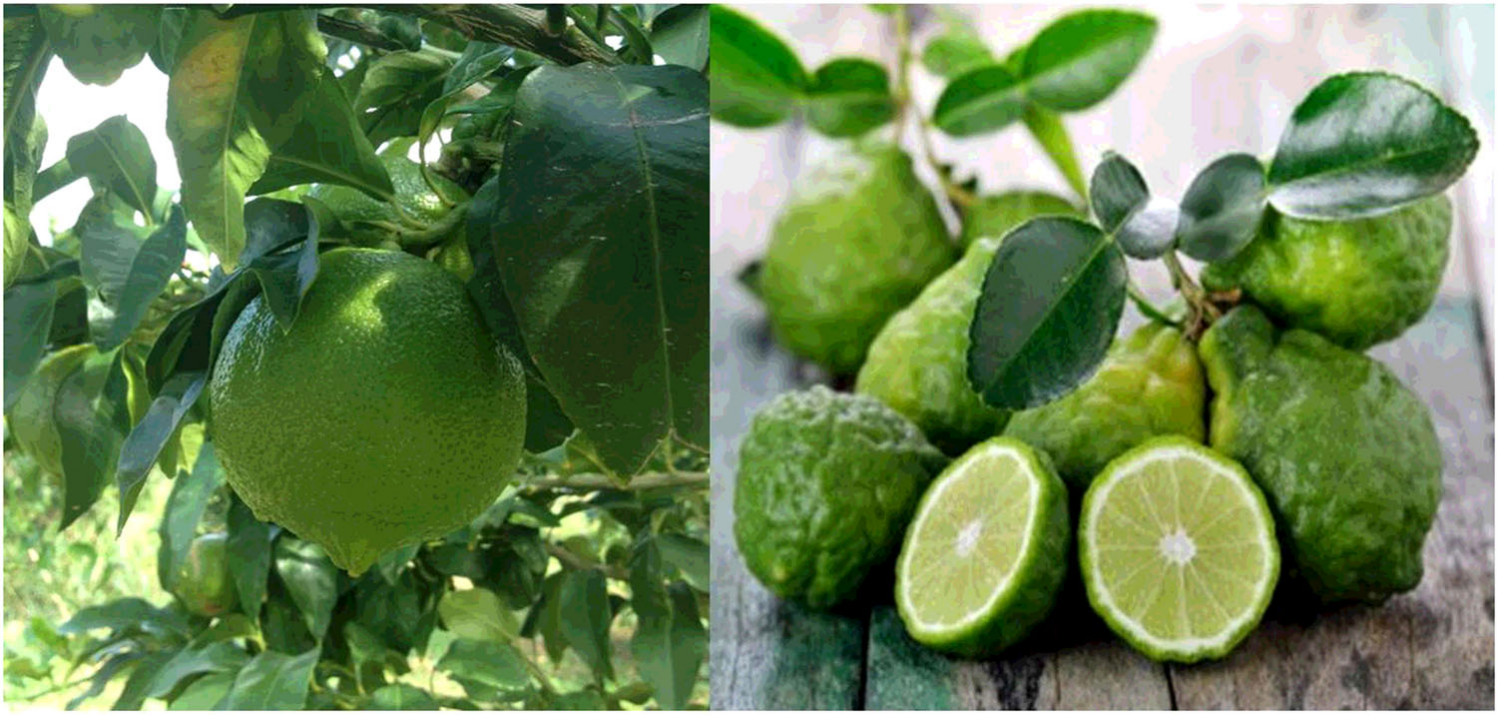
Bergamots from Stefano Bonfà farm of the Samo village in the Reggio Calabria province.

In Italy, TM is also known as folk medicine, and its use has greatly decreased since the 1940s, concurrent with the rise of the chemical and pharmaceutical industry. However, in the last 20 years, there has been renewed interest in TM in Italy and many parts of the world, specifically in relation to medicines based on medicinal plants (Navarra et al. 2015). For example, numerous remedies for the treatment of infectious skin diseases, including anthrax, boils, erysipelas, impetigo, and pustules were developed based on the observation that topical application of fresh plant material has antimicrobial activities (Mazzei et al. 2020). In addition, other plants typically used for non-medicinal purposes, such as *Peristrophe bivalvis* (L.) Merr. (Acanthaceae), whose aqueous leaf extract is used as a non-toxic food dye, have recently been studied for their biological and pharmacological activities (Thuy et al. 2013). Plant-based foods, other than medicinal plants, is also an excellent source of compounds useful for influencing metabolic processes as coadjuvant of pharmacological substances. However, their use as food supplements sometimes requires substantial processing for the production of formulations that possess biological *in vivo* effects. This, for example, is the case of bergamot (*Citrus bergamia* Risso & Poit. [Rutaceae]), a *Citrus* fruit grown on a narrow coastal strip on Calabria, a region of Southern Italy, that contain various components with wide biological activities (Figure 1). Various bergamot formulations containing polyphenols, of which the fruit is rich, have been produced that improve poor gastro-intestinal absorption (Mollace et al. 2019). Additionally, formulations bergamot extracts with those from other foods, such as artichoke (Cicero et al. 2019), wild cardoon (Ferro et al. 2020), or olive (Bonfigli et al. 2020), and/or with pure compounds, such as vitamin K2 (Bonfigli et al. 2020), flavonoids, and pectins (Capomolla et al. 2019), promote positive interactions that could expand the variety of diseases or risk conditions that can be effectively treated.

## History of bergamot

Bergamot is very sensitive to pedoclimatic soil conditions, thus, it grows almost exclusively in a narrow coastal area that extends from Reggio Calabria to Locri in the southernmost part of the Italian peninsula, where 95% of global bergamot production is concentrated. This province has one of the best habitats for bergamot, as it is the only known place where both yield and quality of the essence can be optimized (Navarra et al. 2015). The word bergamot may have been derived from the Turkish word ‘beg-a-mudi’, meaning ‘Pears of the Prince’, based on its close resemblance to the bergamot pear, a fruit shown in a 1715 painting by B. Bimbi. Alternatively, it may originate from the city of Bergamo, where bergamot oil was sold for the first time (Rapisarda and Germanò 2013). The exact origin of this *Citrus* fruit is not known; though the yellow-green color may indicate that it is a derivation by genetic mutation from pre-existing *Citrus* species, such as the sour orange (*Citrus aurantium* L.) and citron (*Citrus medica*). It has been hypothesized that bergamot originated from the Canary Islands, although other sources suggest China, Greece, or the Spanish city of Berga, from which it was transported to Southern Italy (Navarra et al. 2015; Maruca et al. 2017). Due to its particular fragrance, bergamot was initially used primarily by the perfume industry to produce perfumed waters known as ‘bergamot water’ or ‘cologne water’. In addition, it has been utilized for flavoring by the food and confectionery industries and by the pharmaceutical industry to improve the smell of ointments and medicines, as well as for making toothpaste, hair oils, and cosmetic products (Maruca et al. 2017).

Since its introduction to Europe, bergamot essential oil (BEO) has been used in popular medicine, and various curative properties have been attributed to this substance. According to one source from Flückiger and Hanbury (1879), bergamot oil was included in a list of medicines printed in 1688 that were available at a pharmacy in the small German town of Giessen. The first experimental observations on the medicinal and healing properties of bergamot essence were made by Doctor Francesco Calabrò, in the town of Reggio Calabria who, in his 1800 treatise, described its anti-infectious and healing properties. Numerous sources indicate that bergamot was widely used in folk medicine for the treatment of wounds, burns, varicose veins, furunculosis, and toothaches and as an antipyretic/anti-inflammatory agent (Calabrò et al. 1998). In the second half of the eighteenth century, bergamot oil was added in drops to tea as an antimalarial, and it was further administered to treat scabies, as a sedative, and in drops to prevent insomnia (Ferlazzo et al. 2015). In a study from 1932, the surgeon Antonino Spinelli, head of the Ospedali Riuniti di Reggio Calabria, provided *in vitro* and *in vivo* evidence that bergamot may constitute a new antiseptic for surgery due to strong bactericidal activity. More recently, in the province of Reggio Calabria, bergamot has become popular as a natural treatment for the control of cholesterol and triglycerides (TGs).

The beneficial properties of this fruit, which have been observed and applied in folk medicine for several centuries, have attracted renewed attention in recent years. Over the past decade, bergamot has been the subject of substantial modern scientific research and numerous in-depth studies. In this review, we will summarize the major findings from these studies on the antiproliferative and anti-inflammatory effects of bergamot and discuss the implications for current medicine.

## The biology and chemistry of bergamot

**Figure 2.**
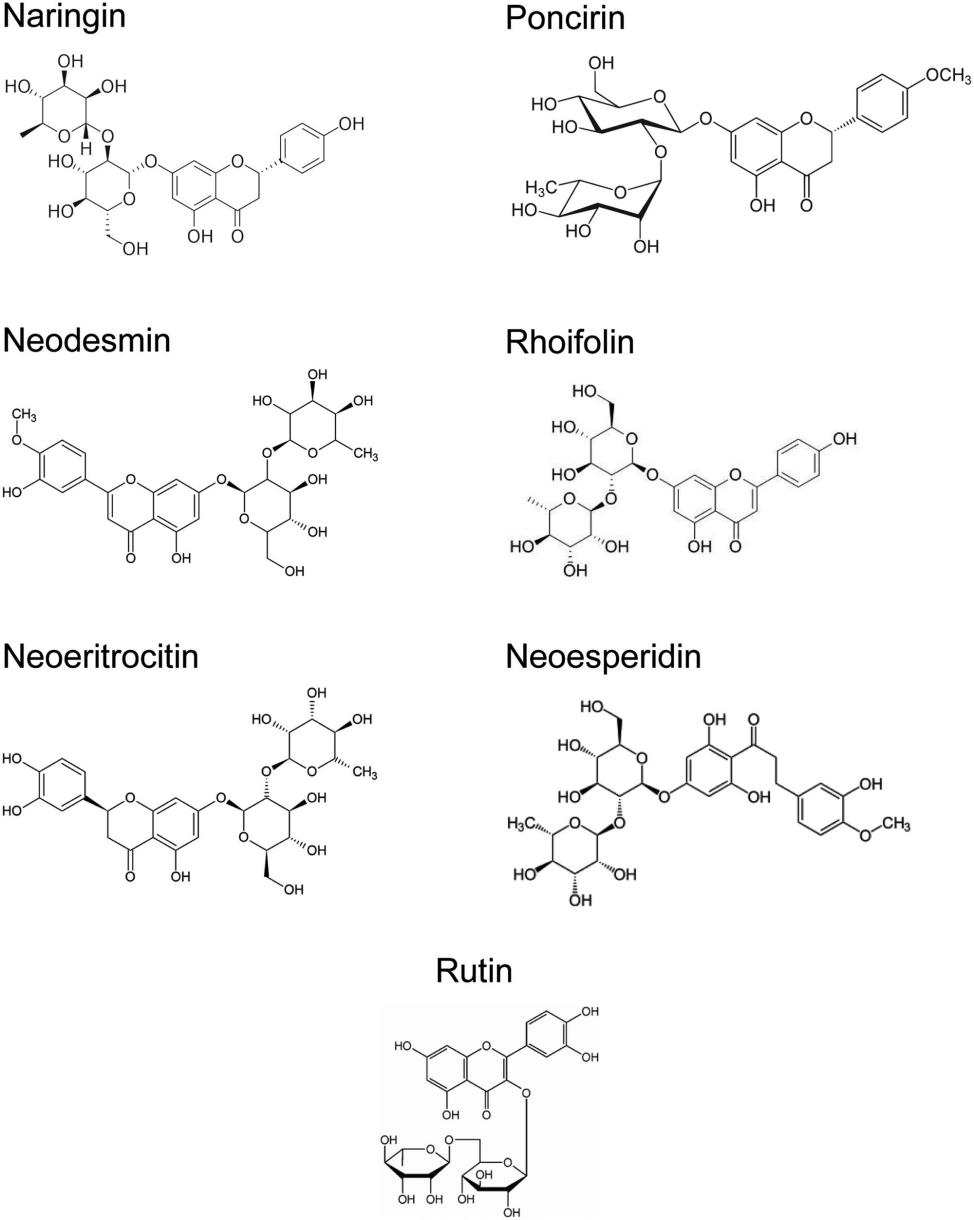
The major flavonoids in BJ and their molecular structures.

The three variants of bergamot –‘fantastico’, ‘feminello’, and ‘castagnaro’- are distinguished based on morphology of the plant and fruit and exhibit distinct aromatic and flavor profiles. As noted above, this ancient plant has long been used for essential oil production from its skin, but recent research has revealed that bergamot fruit pulp also contains high level of phenolic compounds. Bergamot juice (BJ) differs from the juice of other *Citrus* fruits due to its peculiar profile and high content of flavonoids and glycosides (e.g., neoeriocitrin, neohesperidin, naringin, rutin, neodesmin, rhoifolin, and poncirin) (Figure 2), which show a wide range of pharmacological activities (Mondello et al. 1998).

**Figure 3.**
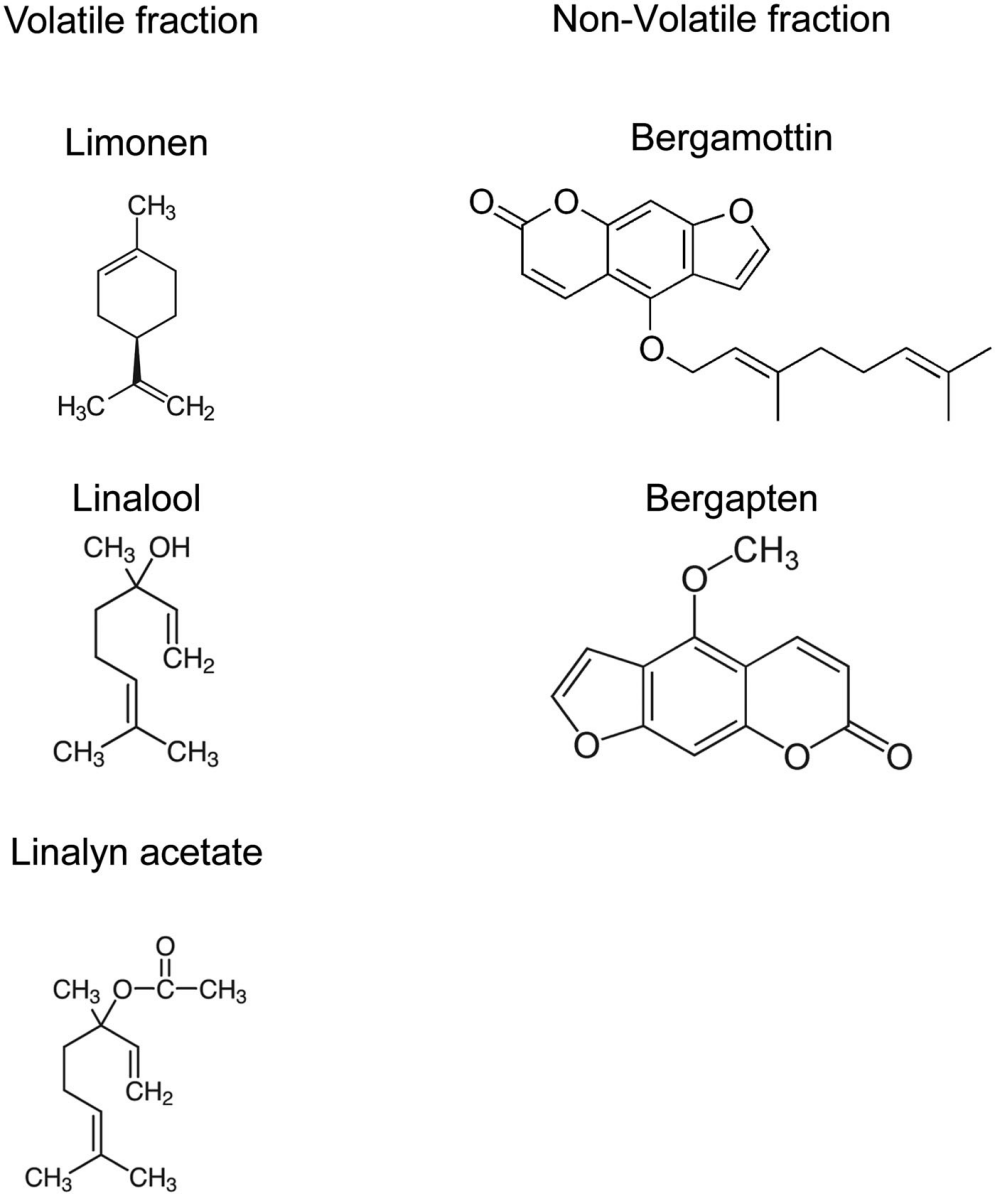
The main constituents of BEO and their molecular structures.

Using high-pressure chromatography and mass spectrophotometry, the phytochemical composition of bergamot has been characterized. In cold-extracted juice and industrial extracts from juice and oil (skins). Quantitative analysis of BEO and BJ revealed these contain a volatile fraction (93–96%), that includes monoterpenic and sesquiterpenic hydrocarbons (25–53% limonene) and oxygenated derivatives (2–20% linalool and 15–40% linalyl acetate) (Verzera et al. 2003; Gattuso et al. 2006; González-Mas et al. 2019). BEO also contains a non-volatile fraction composed of waxes, polymethoxylated flavones, coumarins, and psoralens (e.g., bergapten and bergamottin) (Figure 3).

Linalool and linalyl acetate impart the olfactory notes of the oil or extract, and their content is considered an index of product quality (Statti et al. 2004; Perna et al. 2019). The use of gas chromatography-olfactometry-mass spectrometry has indicated that the distinct smells associated with different mixtures of *Citrus* fruits in black tea and their diverse flavors are due to the presence of compounds such as alkenes that vary according to the species of *Citrus,* and determine its aromatic characteristics (Wang et al. 2020).

The extreme compositional variability observed in the volatile fraction of bergamot oil and extracts results from various factors, including the period of production, cultivar of the fruit, area of origin, and extraction technology. For example, a recent study reported that the screw-press extraction method produces a juice with a higher content of flavanone glycosides than other processes, leading to a higher antioxidant activity, while maintaining the nutritional qualities of fresh-pressed juice (Cautela et al. 2019). Thus, it is important to determine the best cultivation and harvesting conditions Giuffrè and colleagues determined the optimal harvest period for each of the three gene variants to obtain the maximum quantity and quality (Giuffrè 2019).

To optimize the impact of the bergamot products as food supplements, different preparations and formulations must be assessed for their biologically active effects. In addition to BEO (Watanabe et al. 2015), the main preparations commonly used include bergamot extract (BE) (Toth et al. 2015) obtained from BJ and/or fruit pulp, and the bergamot polyphenolic fraction (BPF), which is enriched in polyphenols, such as neoeriocitrin, neohesperidin, and naringin (Toth et al. 2015; Bruno et al. 2017). This last formulation, which has generated considerable nutraceutical and industrial interest, has been widely characterized *via* ultra-performance liquid chromatography with diode array detection mass spectrometry analysis. This technique, complemented by a series of preparative chromatographic separations and nuclear magnetic resonance spectroscopic analysis of individual isolated compounds, has resulted in the identification of new glycosylated flavonoids, such as bergamjuicin (Formisano et al. 2019).

Based on the chemistry of bergamot-associated compounds, new chemical analysis methods, such as gas chromatography, combined with flame ionization detection and gas chromatography combined with mass spectrometry, have been developed. These provide fast, accurate, and sensitive methods for determining the content of every compound present in plant phytocomplexes, such as the amounts of squalene and free sterols (Siano and Cautela 2021). In addition, recent genotyping studies using next-generation sequencing technologies to analyze the genomes of diploid and polyploid organisms of various species within the genus *Citrus* have increased our understanding of their genome complexity and uncovered phylogenetic links between these species. These studies revealed the complex phylogenomic structure of bergamot and indicate that bergamot originates from the hybridization between a sour orange and a lemon (Ahmed et al. 2019).

## Anti-proliferative and pro-apoptotic effects

Numerous *in vitro* and *in vivo* investigations have provided evidence that various forms of bergamot (e.g., extract, juice, essential oil, and polyphenolic fraction) can alter the functionality of several biological pathways, leading to anti-proliferative and pro-apoptotic effects against cancer cells. Apoptosis is a form of programmed cell death and the two main molecular pathways are the extrinsic and the intrinsic pathways. The extrinsic pathway involves stimulation of cell surface death receptors by their ligands; this step is followed by the recruitment of adaptor molecules, that, in turn, recruit and activate caspase-8 (Delfino et al. 2011). The intrinsic pathway is set by the dynamic balance between anti- and pro-apoptotic members of the B-cell lymphoma 2 (Bcl-2) family, followed by the release of cytochrome c from mitochondria and the activation of caspase-9. The two pathways converge at the activation of the terminal/executioner caspase, caspase-3, thus, inducing apoptosis (Muscari et al. 2020).

Several studies have assessed the effect of bergamot extracts on apoptosis and proliferation using various formulations and cancer cell lines. For example, BJE reduces growth rates and apoptosis in colon cancer cells through inhibition of the MAPK pathway and impairment of apoptotic proteins (Visalli et al. 2014), Another study presented potential anti-proliferative properties of BJ in neuroblastoma cells; these effects were due to the high levels of flavonoids contained in bergamot that inhibit the adhesive capacity of cancer cells by affecting actin filaments and the active form of focal adhesion kinase (FAK) and its association to neural cell adhesion molecule (NCAM) (Delle Monache et al. 2013). In addition, BJ shows antiproliferative and pro-apoptotic effects in human hepatocellular carcinoma (HepG2) cells (Ferlazzo et al. 2016) and it inhibits cell grown and adhesion in neuroblastoma cell lines (Navarra et al. 2014). Similarly, these *in vitro* inhibitory effects may underly the observed reduction in lung metastases in response to BE in an *in vivo* mouse xenograft model (Navarra et al. 2014).

Comparison of novel formulations of bergamot have also been tested for possible anticancer activity. In one study, incorporation of BEO into nanoparticles led to an increase in its cytotoxic capacity, compared to free oil at equivalent low concentrations in the Caco-2 CRC tumor cell line (Marchese et al. 2020) indicating a possible therapeutic use in cancer treatment.

Another study found that a mixture of two bergamot-derived flavonoids, brutieridin and melitidin (BMF), acts as a non-toxic inhibitor of mevalonate metabolism and 3-hydroxy-3-methylglutaryl-CoA-reductase (HMGR) in the breast cancer cell lines T47D and MCF7, effectively reducing aldehyde dehydrogenase activity and mammosphere formation. This compound also blocks activation of stem cell-associated signaling pathways, including STAT1/3, Notch, and WNT/beta-catenin, thereby inhibiting Rho-GDI signaling. In addition, high levels of HMGR mRNA in breast cancer patients is associated with poor clinical outcome, suggesting a potential strategy that could be used in conjunction with BMF-mediated personalized therapy (Fiorillo et al. 2018).

**Figure 4.**
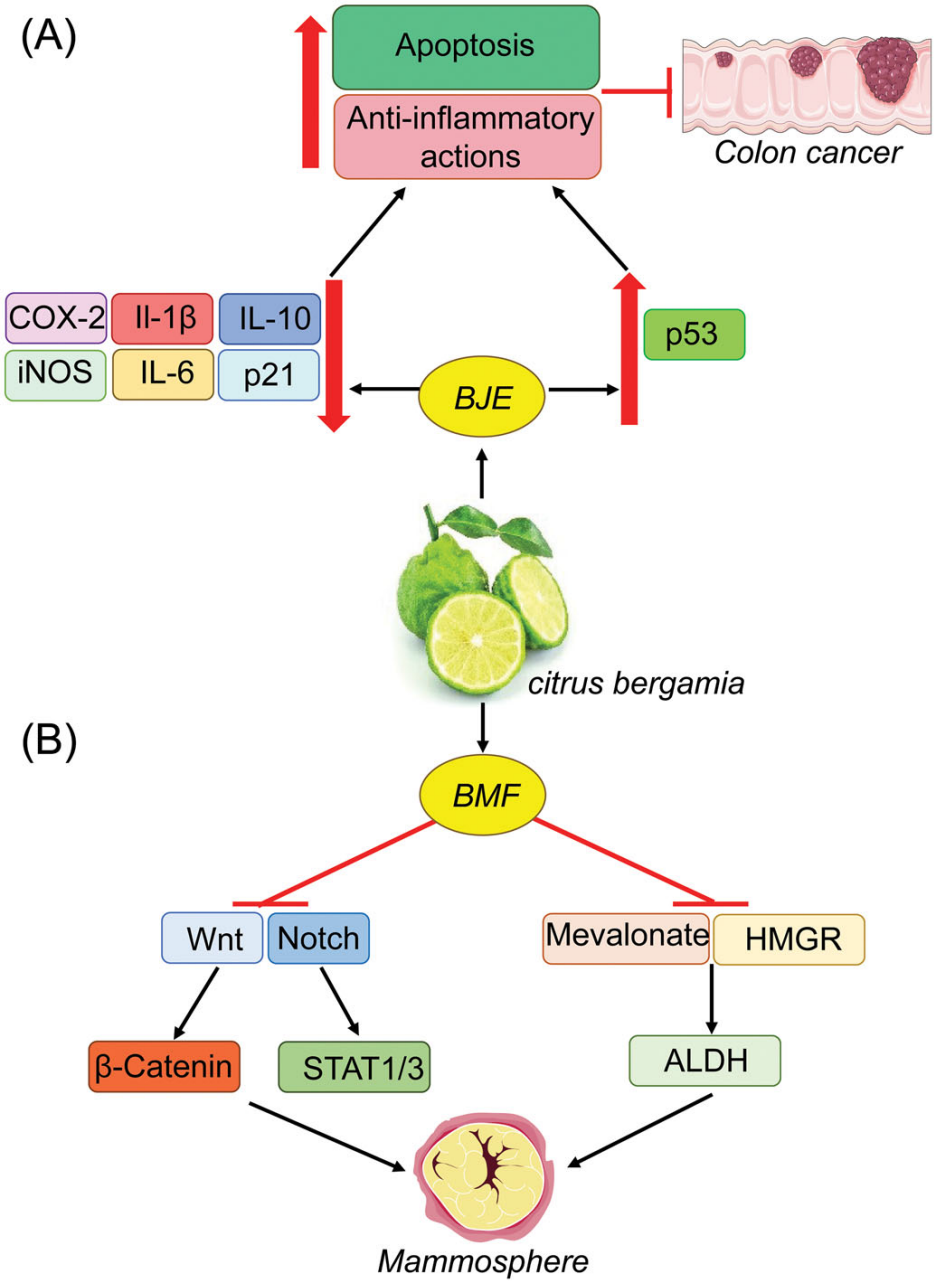
Treatment with BJE downregulated COX-2, iNOS, IL-1β, IL-6, and IL-10 expression in CRC and upregulated p53 expression, apoptosis, and anti-inflammatory activities (A). BMF blocks activation of STAT1/3, Notch, and the WNT/beta-catenin signaling pathway and acts as a non-toxic inhibitor of HMGR to reduce mammosphere formation (B).

Promisingly, one of the first studies performed in an experimental *in vivo* model for CRC (Navarra et al. 2020) showed significant downregulation of inflammation-related genes, including cyclooxygenase-2 (COX-2), nitric oxide synthase, IL-1β, IL-6, IL-10, and arginase, in tumor-bearing rats treated with BJE. Increased apoptosis, upregulation of p53, and downregulation of the survivin and p21 genes were also observed in bergamot-treated animals. These data indicate a strong chemopreventive activity for BJE that is due at least in part to its pro-apoptotic and anti-inflammatory activities. These effects, if confirmed in human studies, have the potential to be exploited, not only as a strategy to prevent CRC in high-risk patients, but also to improve the diagnosis and management of other types of cancer (Figure 4).

Therefore, the *in vitro* and *in vivo* models of different cancer types using various formulations highlight the wide array of pro-apoptotic and antiproliferative effects of bergamot that may be translated into anticancer therapeutics.

## Anti-inflammatory and antioxidant effects

Over the past decade, a renewed interest in natural products as potential sources of drugs and therapeutics has led to investigations into the possible anti-inflammatory activities of BEO, often related to its analgesic activity, in experimental animal models (Karaca et al. 2007; Sharifi-Rad et al. 2017; Dosoky and Setzer 2018; Gandhi et al. 2020). One such study used carrageenan-induced paw edema in rats as an experimental model for inflammation. Due to the toxicity of furocoumarins, a furocoumarin-free fraction of BEO (BEO-FF) was used; pretreatment with this fraction significantly reduces the levels of IL-1β, IL-6, and tumor necrosis factor (TNF)-α levels in inflamed paw homogenates, as well as the nitrite/nitrate and prostaglandin E2 (PGE2) content in paw exudates.

Similarly, the anti-inflammatory activity of BJE was tested in rats with experimental periodontitis induced by a single intra-gingival injection of lipopolysaccharide (LPS). An oral dose of BJE given for 14 consecutive days led to gingival reduction in typical inflammatory markers, including nuclear factor kappa-light-chain-enhancer of activated B cells (NF-κB) translocation and the production of myeloperoxidase and adhesion molecules, such as intercellular adhesion molecule (ICAM) and P-selectin. At the gene level, BJE induced down-regulation of BCL-2-associated X protein (BAX) and up-regulation of B cell lymphoma-2 (BCL-2) expression. Therefore, BJE reduce local tissue damage and may be a novel therapeutic for periodontal diseases (Gugliandolo et al. 2018). Furthermore, due to the different anti-inflammatory mechanisms, these bergamot formulations may increase the anti-inflammatory effects of drugs such as non-steroidal anti-inflammatory drugs (NSAIDs) without the wide array of side effects seen with other drugs such as glucocorticoids.

**Figure 5.**
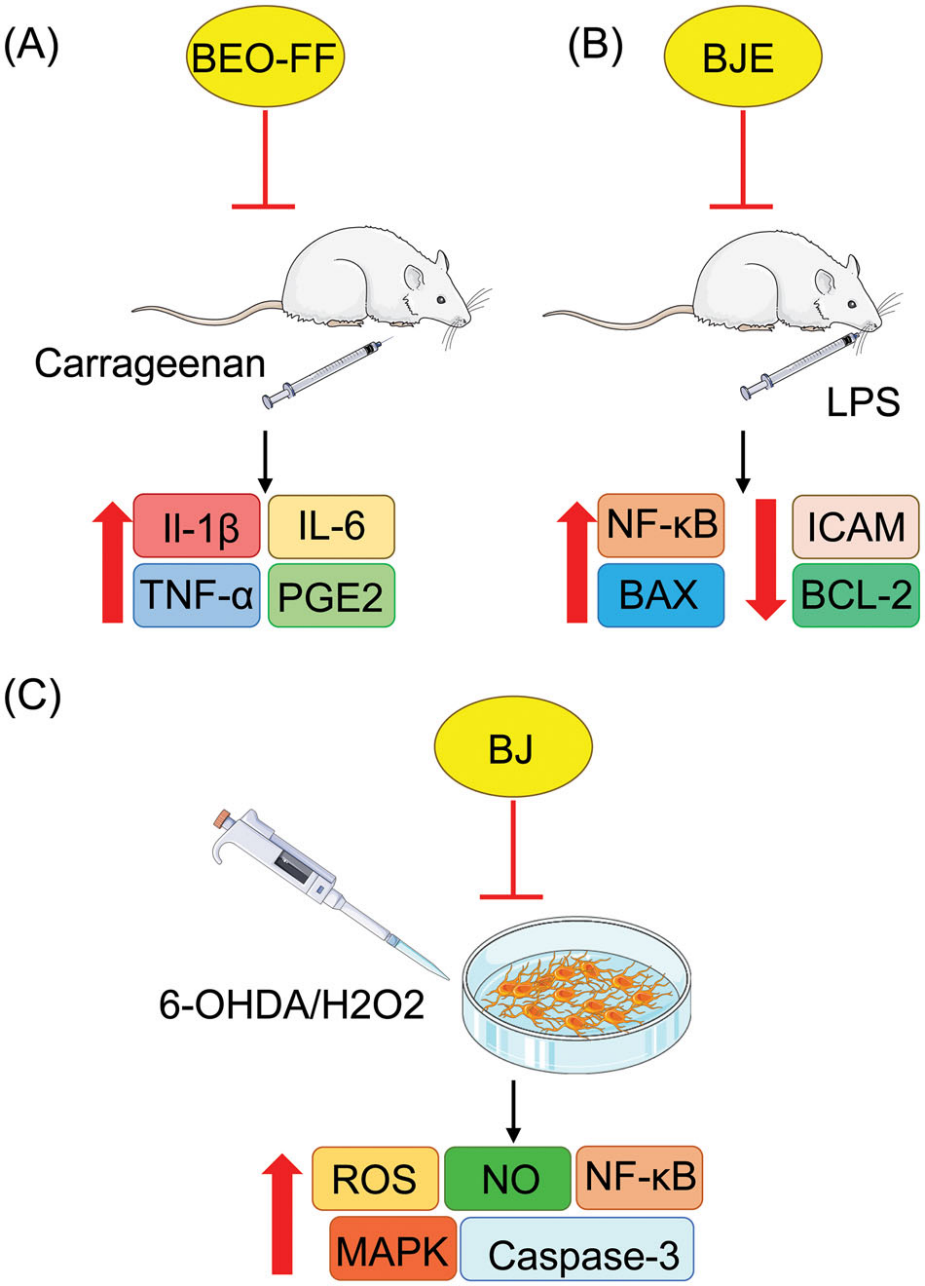
Pretreatment with BEO-FF significantly reduced IL-1β, IL-6, TNF-α, and PGE2 expression levels in paw edema of carrageenan-treated rats (A). An oral dose of BJE reduced NF-κB ICAM, BAX, and BCL-2 expression (B). Human neuroblastoma cells pretreated with BJ show reduced ROS, NO, NF-κB, caspase-3, and MAPK activation (C).

Increasing evidence suggests that both oxidative stress and apoptosis play a key role in the pathogenesis of Parkinson’s disease (PD). Based on the previously reported anti-inflammatory and protective activities of bergamot, one study by Ferlazzo et al. (2020) evaluated whether BJ can exert a protective effect against cell death induced by 6-hydroxydopamine (6-OHDA) or hydrogen peroxide (H_2_O_2_) in neuroblastoma cells. Treatment of differentiated human neuroblastoma cells (SH-SY5Y) with 6-OHDA or H_2_O_2_ resulted in cell death, which was significantly reduced by BJ pretreatment. These protective effects of BJ appear to be related to a reduction in intracellular ROS and NO, which are generated in response to 6-OHDA or H_2_O_2_. BJ also attenuated mitochondrial dysfunction, CASP3 activation, imbalance of pro- and anti-apoptotic proteins, MAPK activation, and NF-κB nuclear translocation, all of which are induced by treatment with the neurotoxic agents. Based on the results obtained from this study, bergamot has an antioxidant capacity that makes it worthy of consideration for the management of neurodegenerative diseases (Figure 5).

Other recent studies have evaluated the anti-inflammatory activity of a new phytochemical formulation called bergapten (Sicari 2018), a furanocoumarin derived from various *Citrus* species (including bergamot) through a complex separation process requiring optimized CO_2_ and temperature conditions. In *in vitro* studies, this phytocomplex decreased the expression and release of pro-inflammatory cytokines, such as TNF-α and interleukins, reduced prostaglandin levels, promote the clearance of neutrophils and macrophages from sites of inflammation, and reduced oxidative stress through the inhibition of ROS (Zhou et al. 2017). In addition, the anti-inflammatory properties of bergapten were further confirmed in a rat acetic acid-colitis model. Following treatment with bergapten, rats with colitis showed a normal colon-weight-length ratio, reduced colon damage, and reduced degranulation of mast cells, which are involved in the inflammatory process, in the mucosa (Adakudugu et al. 2020). Similarly, another bergamot derivative, a heteropolysaccharide termed CMSPB80-1 that was isolated and purified from *Citrus medica* var. *sarcodactylis* by alkaline extraction and characterized by mass spectrometry, shows strong antioxidant activity. This compound enhances the phagocytosis of macrophages and significantly promotes NO production and proliferation of mouse splenocytes, suggesting potential as an immunomodulatory agent (Peng et al. 2019).

The antioxidant and cardioprotective effects of BPF have also been evaluated in the context of cardiac damage induced by doxorubicin (DOXO), common antineoplastic drug. Significant autophagic action was observed in rats treated with DOXO plus BPF, including a significant reduction in cardiomyocytic apoptosis and reactive hypertrophy compared to animals treated with DOXO alone. Thus, integration of bergamot with DOXO treatment counteracts the adverse events associated with DOXO cardiotoxicity by reduction production of ROS and increasing survival of resident endogenous endogenous c-kitpos cardiac stem cells (Carresi et al. 2018).

Recent progress toward understanding the mechanism underlying the anti-inflammatory activity of BJE has revealed that this extract can directly activate the transcription factor NAD-dependent sirtuin-1 deacetylase (SIRT1) (Cantó et al. 2009). A BJE-mediated increase in SIRT1 deacetylase activity *via* a mechanism involving AMPK activation was demonstrated through cell-free *in silico* and *in vitro* experimental models (THP-1 leukemic monocytes exposed to LPS). Consistent with the results described above, these data suggest that bergamot and bergamot derivatives are as promising candidates for the treatment of degenerative pathologies in which the AMPK/SIRT1 axis is compromised, including diabetes, atherosclerosis, and Alzheimer’s disease (Maugeri et al. 2019).

## Conclusions

Bergamot has long been used for many purposes, particularly in the food sector as a flavoring agent and in the cosmetic field for the preparation of scented products, such as deodorants and perfumes (Bruno et al. 2017). In recent years, bergamot has also shown considerable promise for use in a vast array of clinical and pharmaceutical applications. Numerous scientific studies suggest that bergamot formulations can reduce proliferation in a wide variety of cancer cell types *in vitro* by inducing cell cycle arrest. Therefore, bergamot or bergamot derivatives could be used in therapeutic applications as anticarcinogenic agents (Visalli et al. 2014; Fiorillo et al. 2018). The numerous lines of scientific evidence demonstrating the beneficial effects of bergamot represent a starting point for future clinical and molecular studies. It will be extremely important to elucidate the molecular mechanisms that mediate the complex biological properties of bergamot (Table 1).

**Table 1.** Different forms of bergamot tested, models used, effects, and the references.

Different forms of bergamot	Model used	Effects	Reference
Juice	Neuroblastoma cell line HepG2 cells Neuroblastoma cell lines *In vivo* model for CRC Rats with experimental periodontitis induced by LPS Differentiated human neuroblastoma cells (SH-SY5Y)	Anti-proliferative Pro-apoptotic Inhibits cell growth and adhesion Chemopreventive activity, pro-apoptotic and anti-inflammatory activities Anti-inflammatory activities Reduction in intracellular ROS and NO, antioxidant activity	Delle Monache et al. 2013; Visalli et al. 2014 Ferlazzo et al. 2016 Navarra et al. 2014 Navarra et al. 2020 Gugliandolo et al. 2018; Ferlazzo et al. 2020
Essential oil	CRC cells A mouse xenograft model Caco-2 CRC tumor cell line Carrageenan-induced paw edema in rats Rat acetic acid-colitis model THP-1 leukemic monocytes exposed to LPS	Promoting cell death Reduction of lung metastases Increase in its cytotoxic capacity Reduce the levels of IL-1β, IL-6, TNF-α, nitrite/nitrate PGE2 Anti-inflammatory activities Anti-inflammatory activity	Visalli et al. 2014 Navarra et al. 2014 Marchese et al. 2020 Gandhi et al. 2020 Adakudugu et al. 2020 Maugeri et al. 2019
Polyphenolic fraction	T47D and MCF7, cell lines Mouse splenocytes Cardiac damage induced by DOXO in rats	Non-toxic inhibitor of HMGR and mammosphere formation, inhibition Rho-GDI signaling Antioxidant activity Antioxidant and cardioprotective effects	Fiorillo et al. 2018 Peng et al. 2019 Carresi et al. 2018

Research has mainly focused on the pharmacodynamic aspects of bergamot, relating to dosage, time and method of administration, and importantly, type of formulation. Many recent pilot studies have evaluated new bergamot formulations, including novel combinations with various nutraceuticals and new bergamot derivatives; the new formulations can alter product absorption and, therefore, the final effect (Capomolla et al. 2019; Cicero et al. 2019; Mollace et al. 2019; Bonfigli et al. 2020; Ferro et al. 2020).

Characterization and confirmation of the biological effects of bergamot have prompted the use and study of bergamot phytocomplexes in numerous different sectors, resulting in increased demand for bergamot production. Therefore, Calabrian farmers have re-evaluated its potential as an important economic product in the province of Reggio Calabria, where almost all world bergamot production is located. The increasing interest from researchers has yielded numerous experimental and clinical studies that have enhanced the therapeutic profile of bergamot to reveal potential for greater use and a more relevant role in human health. Thus, our view of bergamot today is considerably more complex than that in popular tradition.
